# Threatened Conifers Need Targeted and Integrated Conservation: A Case Study of *Pinus dabeshanensis*

**DOI:** 10.1002/ece3.74580

**Published:** 2026-10-23

**Authors:** Yan Xu, Chao Tan, David Kay Ferguson, Yong Yang

**Affiliations:** ^1^ Co‐Innovation Center for Sustainable Forestry in Southern China, Jiangsu Provincial Key Laboratory for Plant Taxonomy, Resource Conservation and Utilization College of Life Sciences, Nanjing Forestry University Nanjing China; ^2^ Department of Paleontology University of Vienna Vienna Austria

**Keywords:** ex‐situ conservation, genetic diversity, in situ conservation, integrative conservation, *Pinus dabeshanensis*, reintroduction

## Abstract

Due to global warming, over‐exploitation, habitat degradation, environmental pollution, and alien species invasion, around 28% of assessed global species are threatened, with conifers being especially endangered. China has attached great importance to biodiversity conservation, and implemented a series of targeted initiatives over the past two decades to protect and conserve the endangered species in China. *Pinus dabeshanensis* W.C.Cheng et Y.W.Law is a conifer species with an extremely small population and is listed as Grade I in the national key protected wild plants in China. Despite conservation efforts, the effectiveness of these measures remains unknown. In this study, we have assessed the conservation effectiveness of this critically endangered conifer species, and identified conservation gaps to achieve a new conservation plan for the species. This paper should also be helpful for planning conservation of other endangered conifers. We conducted a systematic assessment of the current state of *P. dabeshanensis*, and the effectiveness of in situ and ex‐situ conservation, artificial propagation, and reintroduction based on field investigations and a comprehensive review of the published literature, and thereby identified the conservation gaps. The results suggest that the natural population of *P. dabeshanensis* is extremely small, being composed of only 320 recorded individuals sporadically distributed in Henan, Hubei, and Anhui. Although populations in Yingshan of Hubei, Shangcheng in Henan, and Jinzhai of Anhui are protected in national nature reserves, the largest known wild population in Dawanggou of Yuexi in Anhui is not protected by any nature reserve. Ex‐situ conservation is still at a preliminary stage, with very small numbers planted in botanical gardens and lacking the full range of the species' genetic diversity. We also found that the species suffers from reproductive failure including low pollination success and poor seed set. While reintroduction practices have been conducted at several localities, seedling survival rates vary greatly due to a lack of subsequent management/protection. The lack of standardized reintroduction protocols and management guidelines hinders a consistent and effective implementation of these practices. Although many Chinese institutions including universities, nature reserves, and botanical gardens have been involved in the conservation of *P*. *dabeshanensis*, the overall effectiveness has been limited due to inadequate coordination and fragmented efforts. To substantially advance conservation effectiveness, there needs to be a targeted species conservation plan. Within this framework, it will be necessary to combine different approaches in order to ensure the effective protection of this endangered pine. The present study has involved a comprehensive investigation into the conservation effectiveness of *P*. *dabeshanensis*, identified critical conservation gaps, and proposed an integrated conservation strategy. Our findings and suggestions will also be valuable for the conservation of other endangered gymnosperm species.

## Graphical abstract

This study diagnoses governance fragmentation as a key non‐biological barrier to effective conservation. Secondly, it proposes a targeted, integrated conservation framework that unifies actions of various organizations. Thirdly, it offers a transferable model for conservation of threatened conifer species which will be helpful to improve conservation effectiveness.

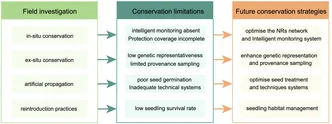

## 1 Introduction

The survival of species has become increasingly problematic. It is estimated that 5% to 15% of global species have already gone extinct (Ceballos et al. 2015). According to the IUCN Red List, 28% of the assessed species are threatened with extinction (IUCN 2024). It is suggested that several factors have been jointly responsible for the loss of global biodiversity since the industrial revolution, including land‐use changes, habitat fragmentation, environmental pollution, climate change, and alien invasive species due to rapid population growth and accelerated urbanization (Newbold et al. 2015; Tilman et al. 2017; Díaz et al. 2019).

Biodiversity conservation has received global attention. In 2022, the 15th Conference of the Parties (COP15) to the Convention on Biological Diversity adopted the Kunming‐Montreal Global Biodiversity Framework, which established the “30 by 30” target—aiming to conserve 30% of terrestrial and marine areas by 2030—alongside other key conservation objectives. This framework now provides a strategic roadmap for global biodiversity conservation efforts, including national initiatives in China (CBD 2022). Furthermore, reducing the rate of biodiversity loss is widely recognized as an urgent international priority (Johnson et al. 2017), with growing evidence demonstrating that targeted conservation interventions can effectively counter projected biodiversity declines (Hoffmann et al. 2010; Zhao et al. 2025). Notably, recovery programs in western Europe have successfully reversed millennia‐long population declines of large carnivores and restored critical habitats for large herbivores, demonstrating the potential for coordinated conservation action to achieve tangible recoveries (Chapron et al. 2014). These collective efforts and emerging successes underscore that while the challenges to global biodiversity are serious, coordinated international frameworks and targeted interventions provide a viable pathway toward reversing current trends and achieving meaningful recovery (Johnson et al. 2017).

The Chinese government has attached great importance to biodiversity conservation and has implemented a series of significant initiatives to reduce the loss of biodiversity. China has played a leading role in proposing and enforcing the ecological conservation redline policy, with designated areas now covering over 30% of the country's total land area (Ministry of Ecology and Environment of China 2022). These measures have effectively protected 90% of terrestrial ecosystem types and 74% of national key protected wildlife species (The State Council Information Office of the People's Republic of China 2021). Over the past two decades, China has launched multiple targeted conservation programs, including the National Project for Rescuing and Conserving Extremely Small Wild Plant Populations (2011–2015) and the ongoing National Construction Plan for Wildlife Conservation Projects (2023–2030) (State Forestry Administration of China 2011; National Forestry and Grassland Administration 2022). The implementation of these initiatives has proved critical for preventing reduction or extinction of wild species populations. Furthermore, to strengthen both in situ and ex‐situ conservation systems, China plans to establish a total of 49 national parks and 10 national botanical gardens by 2035. As biodiversity conservation in China enters a new phase, conducting systematic assessments of conservation effectiveness and identifying existing gaps have become essential for guiding precise, evidence‐based conservation actions.

*Pinus dabeshanensis* W.C.Cheng et Y.W.Law, an endangered species of the family Pinaceae, is endemic to the Dabie Mountains of eastern China (Yu et al. 1978). Xiang (2015) conducted a field investigation of the species and concluded that there were approximately 280 individuals sporadically distributed in the Dabie Mountains, including Yingshan County of Hubei, Yuexi and Jinzhai of Anhui, and Shangcheng in Henan. The species was assessed as Endangered (EN) in the China Biodiversity Red List: Higher Plants (Ministry of Ecology and Environment of China and Chinese Academy of Sciences 2020). This pine species is a flag species in biodiversity conservation in China, being included in the 120 priority species of the National Project for Rescuing and Conserving Extremely Small Wild Plant Populations (2011–2015), and listed among the 50 wild plant species with extremely small populations designated for urgent conservation in the Outline of the 14th Five‐Year Plan for Forestry and Grassland Conservation and Development. This underscores its high scientific value and conservation priority.

Since the launch of the National Project for Rescuing and Conserving Extremely Small Populations of Wild Plants (2011–2015), various institutions and nature reserves have actively implemented actions for the conservation of this threatened pine species. However, due to a lack of fresh investigations, it remains unclear whether the endangered status of this pine species has improved and whether the conservation actions were effective or not. A systematic assessment of the current status and conservation gaps of *P*. *dabeshanensis* is crucial for both developing new conservation plans and improving conservation effectiveness.

In this study, we conducted investigations of *P*. *dabeshanensis* in its natural habitat and collected conservation data, which was supplemented by surveying online databases, reviewing published literature, and unpublished scientific expedition reports of nature reserves. By doing this, we aimed to systematically evaluate the present status of the species, identify conservation gaps, clarify threats and challenges, and develop an integrated conservation framework. Our study will lay a solid basis for guiding future conservation actions.

## 2 Materials and Methods

To understand the research gains and the conservation effectiveness of *P. dabeshanensis*, we established a dataset encompassing a number of fields, including distribution, population size, threats, in situ and ex‐situ conservation status, artificial propagation status, and reintroduction practices.

We compiled this conservation dataset using available online databases including the Plant Photo Bank of China (PPBC, https://ppbc.iplant.cn/), the Global Biodiversity Information Facility (GBIF, https://www.gbif.org/), and the Chinese Virtual Herbarium (CVH, http://www.cvh.ac.cn/), as well as data from published literature and regional floristic works. All data and management details presented in this study regarding in situ and ex‐situ conservation, reintroduction, and artificial propagation were obtained from field investigations, scientific expedition reports, conservation planning documents, feedbacks from nature reserves, and expert consultation.

We conducted a field investigation to assess the current conservation status of this pine species. Field investigations were carried out across the distribution range of *P*. *dabeshanensis*, including the Henan Dabie Mountain and Liankang Mountain National Nature Reserves, the Hubei Dabie Mountain National Nature Reserve, the Anhui Yaoluoping National Nature Reserve, and Dawanggou of Yuexi County in Anhui. We compiled statistics on the basic characteristics of this plant species, such as its population size and age structure, by combining on‐site investigations with standardized interviews and questionnaire surveys. Subsequently, we examined in situ conservation of *P. dabeshanensis* within its distribution range, including population size of each stand, the location of nature reserves, habitat conditions, and current protective measures; artificial propagation, including location of breeding base, scale, number of propagated seedlings, seed source, and seedling survival ratio; and reintroduction practices, including reintroduction sites, number of reintroduced individuals, and survival ratio. Additionally, information about in situ conservation in Jinzhai County of Anhui was collected through consultation with experts. Information regarding ex‐situ conservation was obtained from a survey of botanical gardens, the China Big Data Platform for Ex‐situ Plant Conservation, and consultation with specialists. All field conservation investigation work in this study strictly complied with national laws and regulations concerning wild plant conservation, and research permits were obtained from the corresponding competent authorities.

## 3 Results

### 3.1 Population Status of *P*. *dabeshanensis*

The natural population of *P*. *dabeshanensis* comprises 320 individuals that are distributed in three adjacent provinces (Figure 1, Table 1). Population size varies greatly between different localities. The largest population of the species, which exists in Dawanggou (Yuexi, Anhui), consists of 225 individuals, i.e., ca. 70.53% of the total natural population. Other populations of this species are sporadically distributed in the following locations: Henan Dabie Mountain National Nature Reserve in Shangcheng County, Henan Province (48 individuals); Tianma National Nature Reserve in Jinzhai County, Anhui Province (30 individuals, including 11 seedlings); Hubei Dabie Mountain National Nature Reserve in Yingshan County, Hubei Province (16 individuals, including 13 seedlings). In addition, one wild individual still exists in Yaoluoping National Nature Reserve in Yuexi County, Anhui Province, being the tree from which the type specimen of the species was collected. The natural populations exhibited an obvious declining trend characteristic of an inverted pyramid age structure, and consisted mainly of mature and senescent trees with problematic population regeneration under natural conditions (Xiang et al. 2016).

**FIGURE 1:**
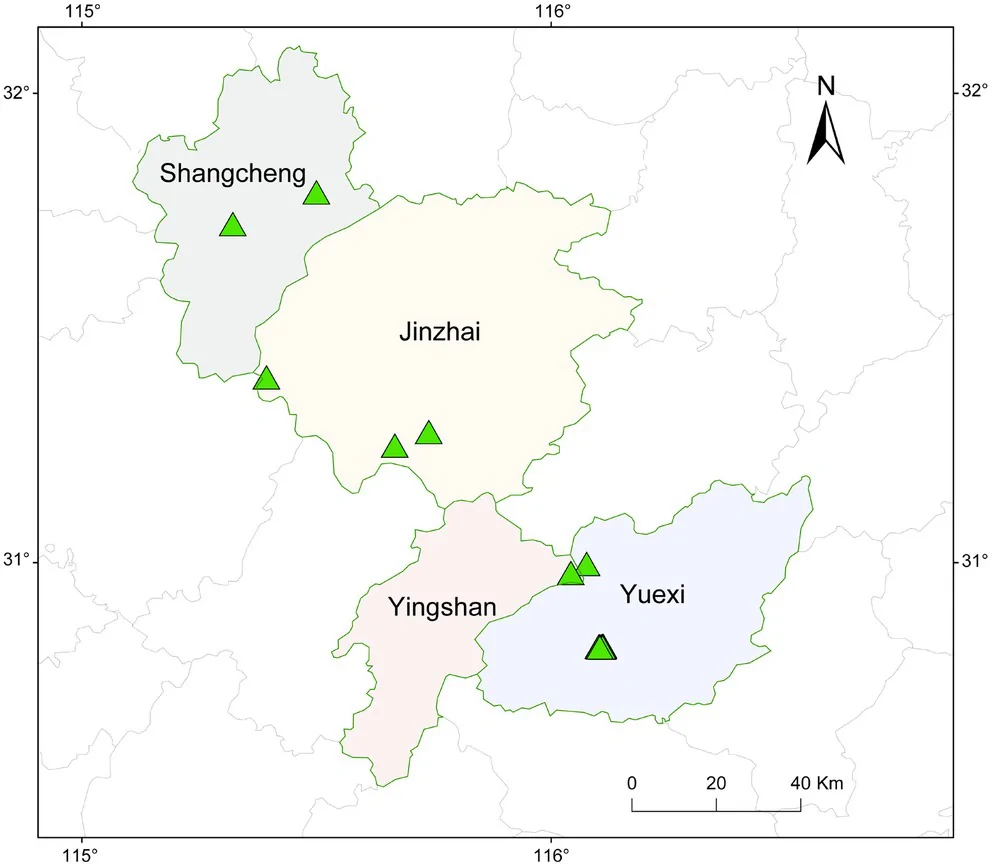
Natural distribution of *P*. *dabeshanensis*. The “
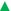
” symbol indicates the occurrence of wild populations of *P*. *dabeshanensis*.

**TABLE 1:** Conservation status of *P. dabeshanensis* in each distribution area.

Site	Distribution area	Total wild abundance	In situ	Artificial propagation	Reintroduction (Survival ratio)	Limitations	Data source
Shang cheng	Henan Dabie Mountain National Nature Reserve	48	48	800	2500 (> 24%; Dawangzi > 60%)	Low seed germination rate	Henan Dabie Mountain National Nature Reserve; Field investigation
Xin	Henan Liankang Mountain National Nature Reserve	/	/	/	200 (10%)	Low seedling survival rate	Field investigation
Ying shan	Hubei Dabie Mountain National Nature Reserve	16	16	2000	> 1300 (> 80%)	Intelligent monitoring system has not yet been established	Field investigation
Yuexi1	Anhui Yaoluoping National Nature Reserve	1	1	200	/	Severe pest infestation and serious senescence of type tree	Field investigation
Yuexi2	Dawanggou	225	/	8000	> 2000 (> 60%)	Outside protected areas and face severe threats	Field investigation; Xiang 2015
Jinzhai	Tianma National Nature Reserve	30	30	/	/	/	Tianma National Nature Reserve

### 3.2 Conservation Effectiveness of *P*. *dabeshanensis*

#### 3.2.1 In Situ Conservation Status of *P*. *dabeshanensis*

A preliminary in situ conservation network for *P*. *dabeshanensis* has been established. First, most natural stands of the species fall within national nature reserves (Figures 2 & 3), with only the Dawanggou population of Yuexi County in Anhui growing outside any known nature reserve (Figure 4). Though a local protected area was established to safeguard the Dawanggou population, no special efforts have been made for an in situ conservation of the population (Figure 4). Second, the Henan Dabie Mountain National Nature Reserve, the Henan Liankang Mountain National Nature Reserve, and the Anhui Yaoluoping National Nature Reserves have developed intelligent monitoring systems equipped with infrared cameras, real‐time video tracking devices, and ecological factor data collection facilities. The Hubei Dabie Mountain National Nature Reserve is not equipped with an intelligent monitoring system due to a lack of additional financial support. Third, habitat management was demonstrated to be effective for in situ conservation of *P. dabeshanensis*. The Hubei Dabie Mountain National Nature Reserve implemented targeted conservation for the three mature plants of the pine species by erecting a surrounding fence to isolate the three plants from human disturbance, installing supportive steel frames to prevent old plants from being blown over, backfilling soil to stabilize the root systems, conducting cavity disinfection and restoration by filling the hollow trees with a professional tree wound sealant, controlling pests and diseases with pesticides, and removing the shrubs and herbaceous plants and litter close to the pine trees (Figure 2A–C). These actions not only successfully protected the target trees but also facilitated seed germination and seedling growth in the vicinity of the mother trees (Figure 2D).

**FIGURE 2:**
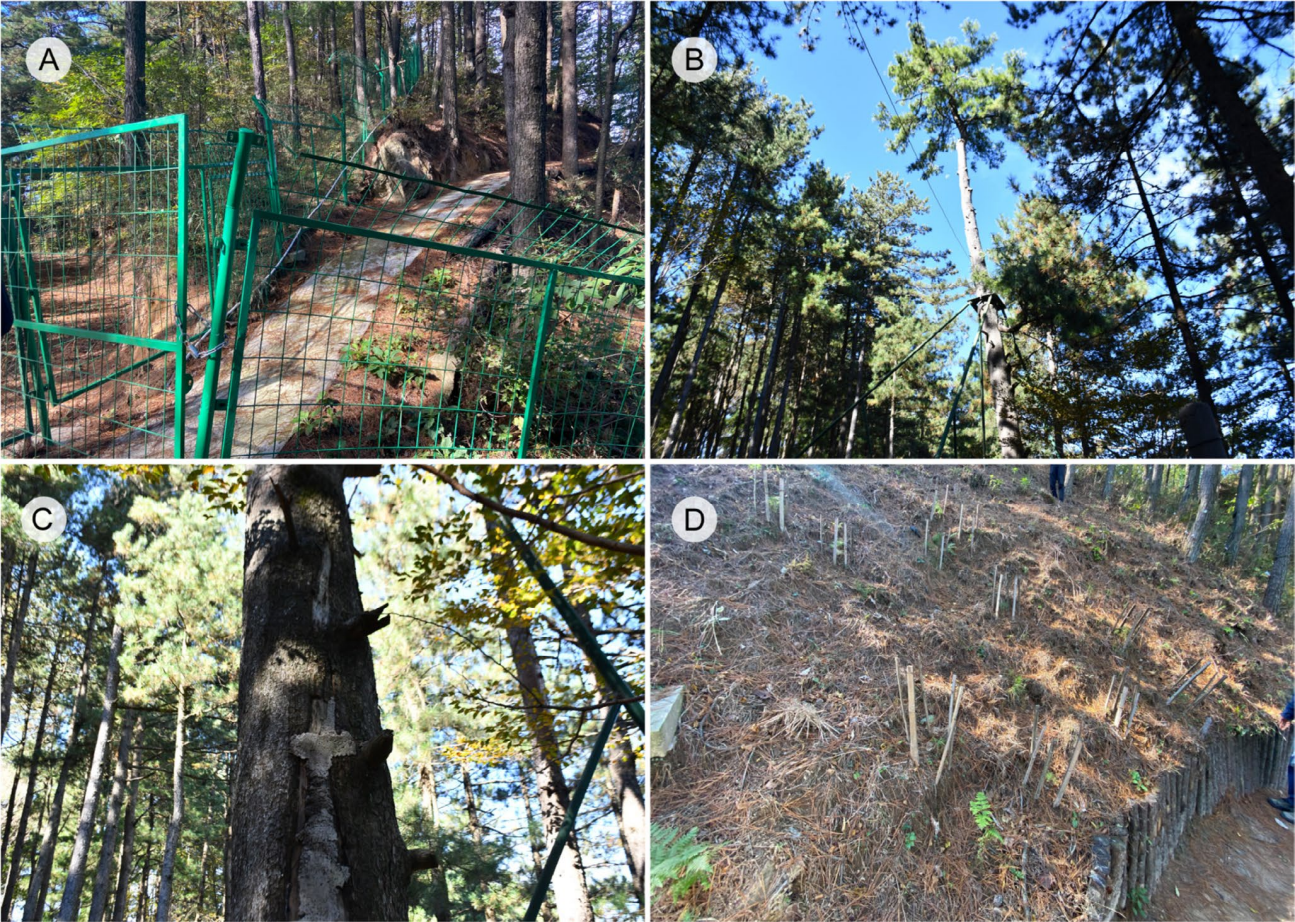
In situ conservation of *P*. *dabeshanensis* in the Hubei Dabie Mountain National Nature Reserve. (A) The protective fence isolating the three trees of *P*. *dabeshanensis* from human disturbance; (B) Supportive steel structures to prevent an old pine tree from falling; (C) Disinfection of damaged tree cavities followed by cement infilling to prevent decay and disease infection; (D) Establishment of bamboo enclosures for the 14 seedlings growing close to the No. 2 mother tree to protect them from animal predation. Photos by Chao Tan & Yan Xu.

*Pinus dabeshanensis* is currently threatened by pests and diseases, the control of which requires improvement. The Hubei Dabie Mountain National Nature Reserve has actively implemented pest‐ and disease control for *P*. *dabeshanensis*, and thus achieved a certain amount of success. In the station, routine pesticide spraying and trunk injection were used to control pests affecting *P*. *dabeshanensis*. Moreover, for hollow trunks, disinfection treatment and cement casting techniques were adopted (Figure 2C). It transpired that these conservation actions were effective in the protection of the three adult trees, as they continue to grow successfully.

The type tree in the Anhui Yaoluoping National Nature Reserve is diseased (Figure 3A–C), so the reserve implemented multiple conservation actions to save the tree. Firstly, the tree was diagnosed as affected with Pine Needle Blight and Pine Rust. In the initial stage of Pine Rust, the tree was sprayed with Amobam 50% SC (diluted 100‐fold), a Thiram/Ziram mixture 50% WP (diluted 500 to 1000‐fold). For Pine Needle Blight, withered and diseased branches were promptly pruned, followed by three consecutive applications of a 100‐fold dilution of Calcium sulfate. Secondly, *Diprion nanhuaensis* sawflies severely damaged the needles of this pine. To mitigate and eliminate the influence of *D. nanhuaensis* larvae, the type tree was sprayed with pesticide. Thirdly, the type tree was also diagnosed as affected by frost damage. To solve this problem, the reserve implemented protective measures including applying anti‐freeze solution diluted 300–450 times to the soil surface, laying straw mats over the root zone to prevent main root frost injury, spraying the trunk with anti‐freeze solution at a dilution of 100–150 times, and wrapping the trunk with geotextile fabric to protect it from the cold (Figure 3D–E). The nature reserve also undertook aeration of the soil around the type tree and applied fungicides to eliminate pathogens and pests in the soil, aiming to provide the root system of the type tree with a favorable and secure soil environment. Fifthly, the tree wounds were disinfected, followed by the application of a conifer‐specific wound sealant. This sealant solidified the resin, thereby reducing nutrient loss (Figure 3E). Despite these conservation actions, the health of the type tree had not improved at the time of our field investigation in October 2024; the tree crown was dead with no needles left. In early October of 2025, we were informed that the situation was getting even worse, as most branches of the type tree had lost their needles.

**FIGURE 3:**
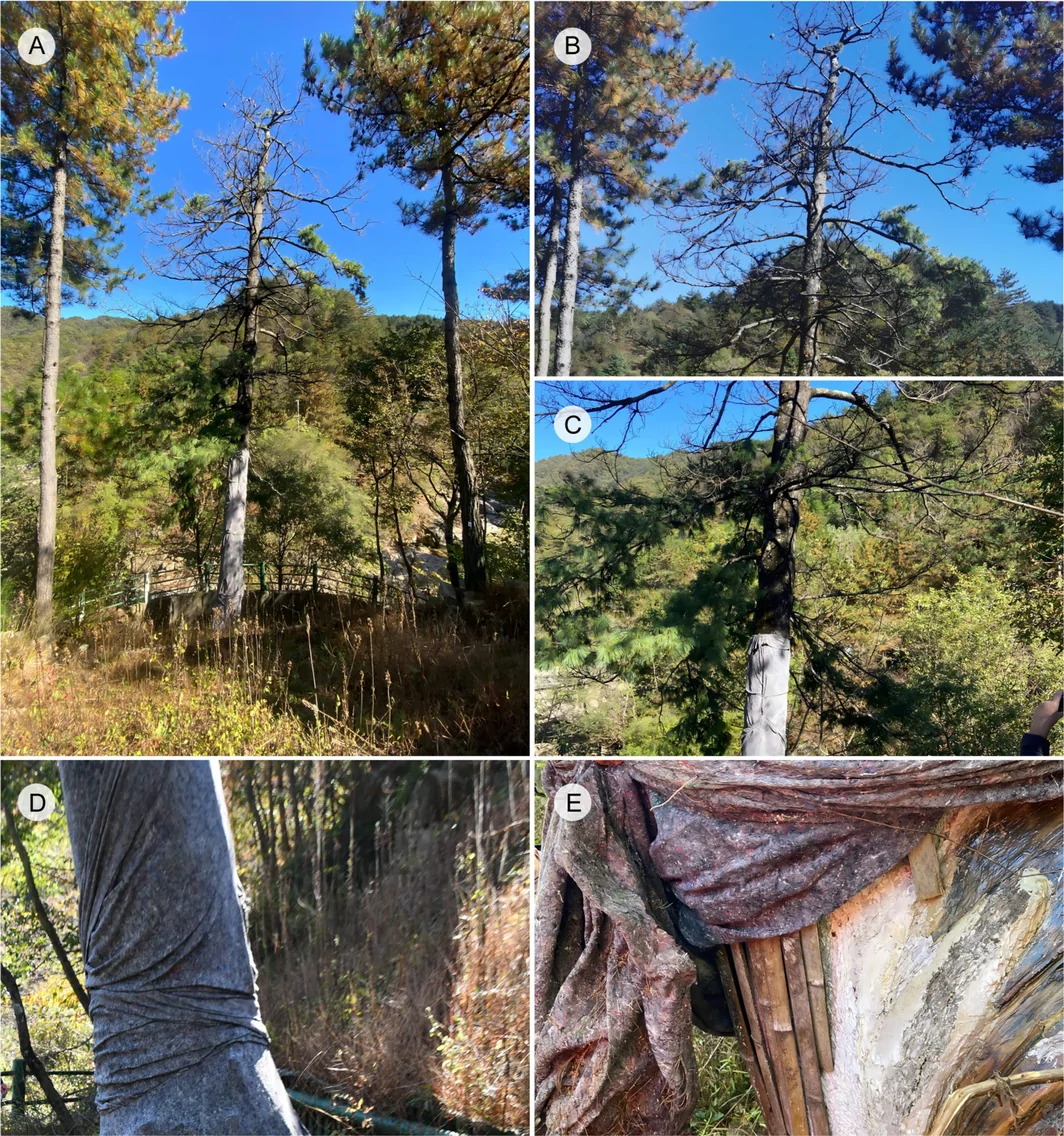
Conservation of the type tree of *P*. *dabeshanensis* in Anhui Yaoluoping National Nature Reserve. (A) Habit of the type tree; (B) Upper portion of the type tree displaying the withered crown in October 2024; (C) Lower portion of the type tree displaying the normal branches in October 2024; (D) Thermal protection wrapped round the trunk of the type tree; (E) Wound restoration treatments implemented on the type tree. Photos by Chao Tan & Yan Xu.

**FIGURE 4:**
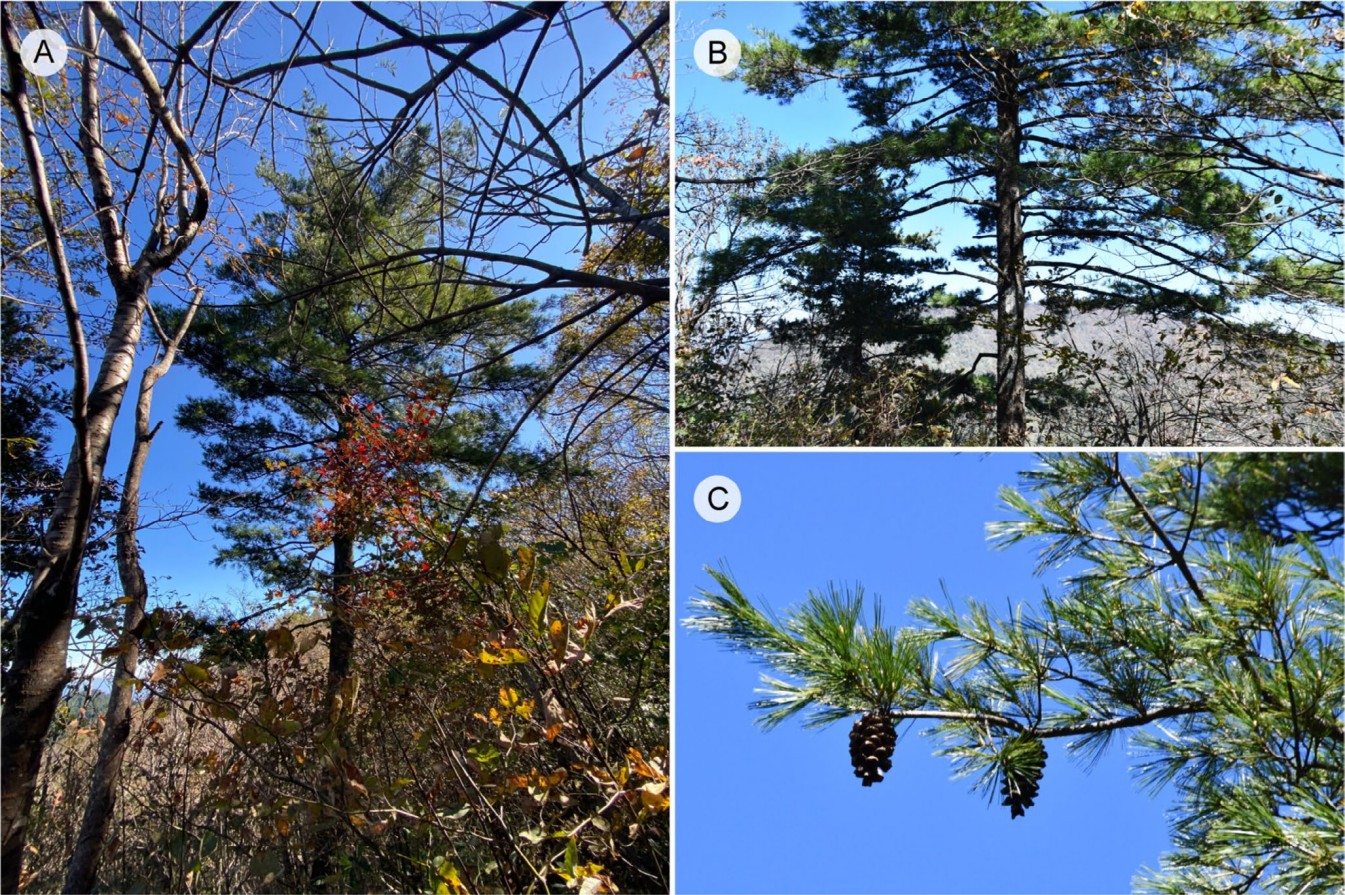
Status of the natural population of *P*. *dabeshanensis* in Dawanggou, Yuexi County, Anhui Province. (A) The entire plant of *P*. *dabeshanensis* and its habitat in Dawanggou. (B) Detail of the tree displaying branches and trunk. (C) A branch of the tree displaying female cones. Photos by Chao Tan & Yan Xu.

#### 3.2.2 Artificial Propagation and Reintroduction of *P*. *Dabeshanensis*

Artificial propagation has been undertaken by the Henan Dabie Mountain National Nature Reserve, the Hubei Dabie Mountain National Nature Reserve, and a local farmer at Miaodaoshan Village in Dawanggou (Table 1, Figure 5A–I). The Miaodaoshan farmer had long been engaged in propagating *P*. *dabeshanensis* using seeds and established the largest nursery for this pine species. He and his family cultivated over 8000 saplings varying from two to seven years (Table 1, Figure 5D–E). The Hubei Dabie Mountain National Nature Reserve established a nursery of 38‐ha for the propagation of the pine species, planting more than 2000 seedlings and saplings (Table 1, Figure 5A–C). Since 2011, the Henan Dabie Mountain National Nature Reserve, working together with the Xinyang Forestry Research Institute, has raised ca. 800 saplings from seed (Figure 5H–I); these were reintroduced into the Xihe Forest Area of the reserve (Table 1, Figure 6G–H). The Anhui Yaoluoping National Nature Reserve propagated over 200 saplings (Table 1, Figure 5F–G). Active propagation efforts of *P*. *dabeshanensis* were made in Henan, Hubei, and Anhui, forming a preliminary network. These efforts have not only increased the number of living individuals of this pine species, but also laid a crucial foundation for reintroduction.

**FIGURE 5:**
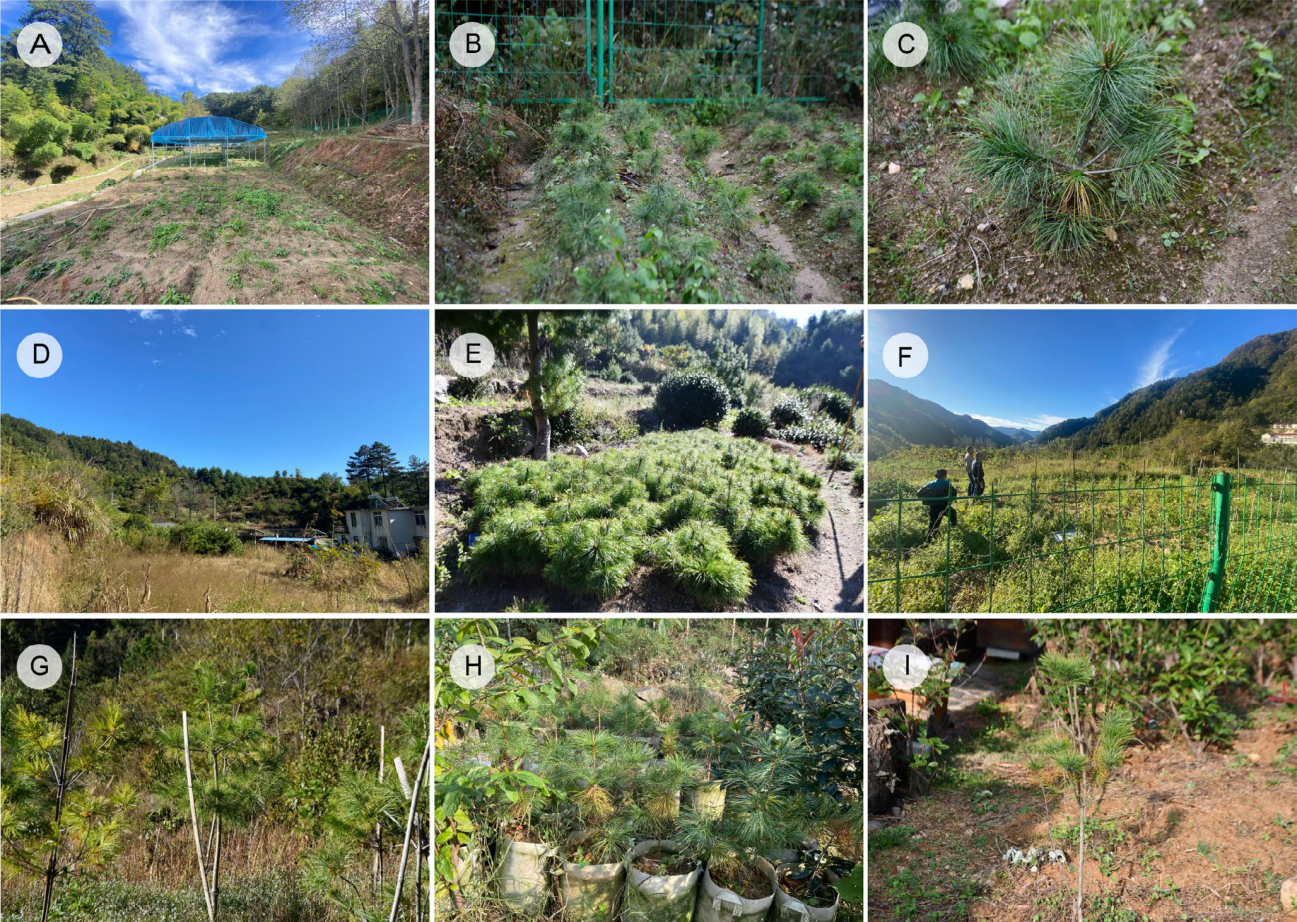
Assisted propagation of *P*. *dabeshanensis* in different nature reserves. (A) The nursery of the Hubei Dabie Mountain National Nature Reserve. (B, C) Seedlings of *P*. *dabeshanensis* propagated from seeds in the nursery of the Hubei Dabie Mountain National Nature Reserve. (D, E) The propagation nursery of Miaodaoshan Village, Yuexi County, Anhui; seedlings propagated from seeds. (F, G) The nursery of the Anhui Yaoluoping National Nature Reserve; seedlings propagated from seeds introduced from Miaodaoshan Village. (H, I) Seedlings propagated from seeds in the Henan Dabie Mountain National Nature Reserve. Photos by Chao Tan & Yan Xu.

**FIGURE 6:**
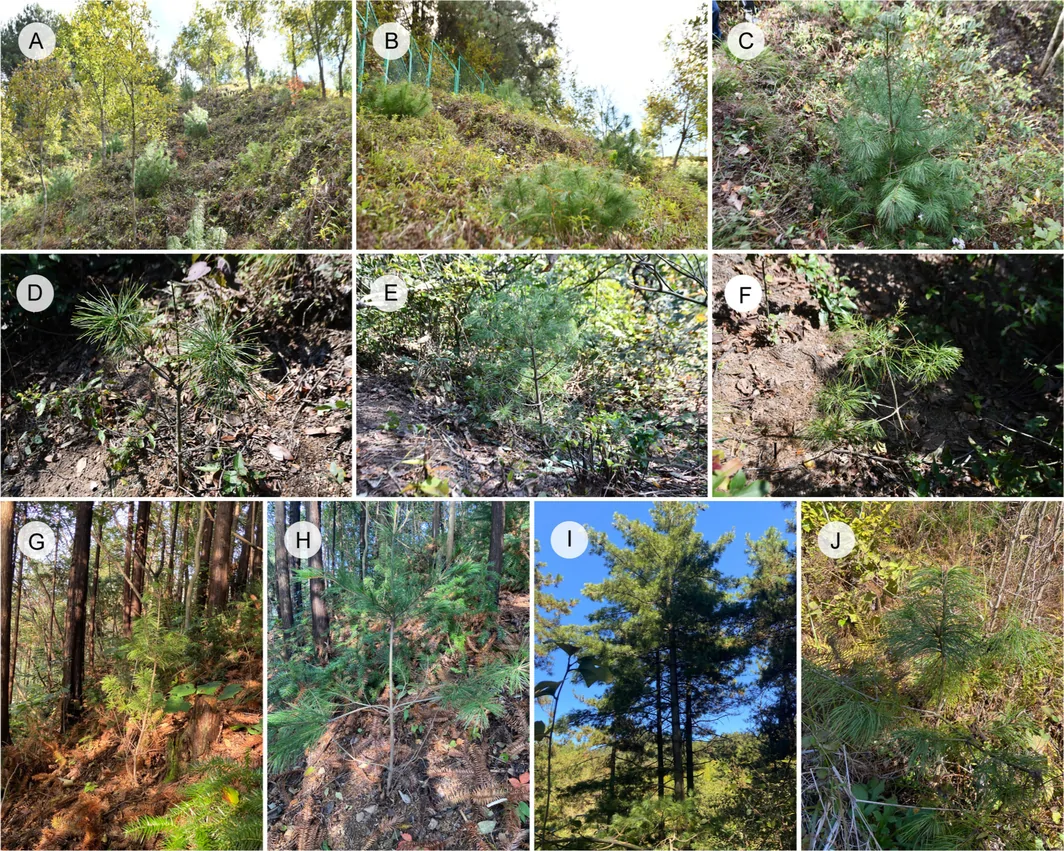
Reintroduction of *P*. *dabeshanensis*. (A–C) Saplings reintroduced into forest land in the Hubei Dabie Mountain National Nature Reserve. Some trees were retained for shading and protective fencing installed around the reintroduction site. (D, F) Saplings at the reintroduction site in the Henan Liankang Mountain National Nature Reserve. No artificial management was implemented, and the growth vigor was weaker compared with the Hubei population. (G, H) Reintroduced saplings in the Dawanzi area of the Henan Dabie Mountain National Nature Reserve. (I, J) Reintroduced saplings at the Wenao Forest Farm in Dawanggou, Yuexi County, Anhui Province. Photos by Chao Tan & Yan Xu.

*Pinus dabeshanensis* was reintroduced to its natural range and nearby localities in Henan, Hubei and Anhui (Figure 6A–J). The Wenao Forest Farm of Dawanggou (Yuexi, Anhui) conducted the earliest reintroduction of this pine species. Since 1984, the Yuexi Forestry Bureau, working with local farmers, undertook reintroduction on the forest farm, planting over 2000 saplings in 40 years, with a survival rate exceeding 60% (Table 1, Figure 6I–J). The Hubei Dabie Mountain National Nature Reserve also undertook reintroduction in recent years and transplanted more than 1300 saplings into forest habitats close to the three mother trees, with a survival rate of over 80%. This represents the highest survival rate of reintroduced saplings of *P*. *dabeshanensis* thus far (Table 1, Figure 6A–C). Reintroduction efforts were also implemented in the Henan Dabie Mountain National Nature Reserve and the Liankang Mountain National Nature Reserve. In the Henan Dabie Mountain National Nature Reserve, a total of 2500 saplings have been reintroduced to nearby forest areas since 2011, with a survival rate of 24% (Table 1). At one of the localities (Dawanzi), most of the established saplings are growing well, with an average annual growth of 5–10 cm (Figure 6G–H). Besides the Dawanzi locality, there are two other relatively large plots in the reserve: 55 seedlings and saplings are being grown in the Rare and Endangered Botanical Garden of the Xihe Protection Station, with another 140 saplings established in a separate locality (Maquewan of Xihe Forest Area). Additionally, five smaller reintroduced populations, each consisting of 10–15 individuals, are maintained in Maoershi, Huangdian, Chaqijian, Pingdingpu, and Taiyangjian, respectively. Staff of the Liankang Mountain National Nature Reserve obtained 200 saplings from Dawanggou and reintroduced them into local forests, with a survival rate of no more than 10% (Figure 6D–F). Meanwhile, the Anhui Yaoluoping National Nature Reserve has planted saplings and is planning to conduct reintroduction in the near future. Among these practices, the Hubei Dabie Mountain National Nature Reserve has done an excellent job, having established a nursery for this pine species, reintroducing 3–5‐year‐old saplings to shady or semi‐shady slopes in the natural distribution range of the target pine species, and improving the survival rate after reintroduction by lowering interspecific competition, a highly effective measure.

#### 3.2.3 Ex‐Situ Conservation of *P*. *dabeshanensis*

Our consultations show that several botanical gardens in China have conducted ex‐situ conservation of this pine species, these include Chenshan Botanical Garden (Shanghai), Emeishan Botanical Garden (Sichuan), Guilin Botanical Garden (Guangxi), Qinling Botanical Garden (Shaanxi), Shanghai Botanical Garden (Shanghai), Wuhan Botanical Garden (Hubei), and Zhongshan Botanical Garden (Jiangsu). All introduced seeds were sourced exclusively from the Dawanggou population of Yuexi in Anhui. According to Zhan (2022), a total of 131 individuals were introduced to Chinese botanical gardens, with 88 surviving to date, representing a survival rate of 67.2%.

## 4 Discussion

### 4.1 Population Status of *P. dabeshanensis* and Driving Factors

Our findings found that the current population of *P. dabeshanensis* is small, a status that may be attributable to multiple factors. The Dabie Mountains are the primary distribution area for *P. dabeshanensis*, yet the natural environment in some parts of the region has been affected by human activities. As *P. dabeshanensis* possesses extremely high ornamental value, some individuals have been illegally dug up and sold; at the same time, indiscriminate logging and development projects have led to the fragmentation of its habitat, resulting in a continuous population decline (Xu 2016; Yan et al. 2024).

Previous studies have found that some gymnosperms suffer from reproductive difficulties; for example, *Pinus squamata* and *Abies beshanzuensis* have very low seed‐setting rates due to difficulties with pollination and fertilization, resulting in very few wild individuals (Su et al. 2021). This phenomenon is also observed in *P. dabeshanensis*. As a wind‐pollinated plant, its pollen dispersal capacity declines significantly at distances exceeding 50 m; furthermore, the high canopy closure density, coupled with rainy and foggy weather conditions, hinders wind‐mediated pollen dispersal, thereby resulting in a low seed‐setting rate (Xiang et al. 2014).

Seed predation by animals is a major factor contributing to the difficulties faced by some gymnosperms during natural regeneration (Su et al. 2018; Ding et al. 2024). For example, *Torreya fargesii* has a low seed‐setting rate and its seeds are readily devoured by forest animals such as flying squirrels, resulting in an extremely low yield of mature seeds (Jiang et al. 2018). *P*. *dabeshanensis* possesses only limited seed dispersal capability. The seeds depend primarily on animal dispersal due to the lack of effective wind‐dispersal structures (such as a seed wing). Moreover, animals consume or hoard the seeds, restricting the dispersal distance and reducing the dispersal of seeds to open habitats where new populations could become established. This has exacerbated the decline in the species' population (Su et al. 2018).

Climate change exerts significant impacts on natural ecosystems and biodiversity, and has become a major threat to species survival (Chen and Li 2004; Xu et al. 2023; Harrison et al. 2020). Due to global warming and the high sensitivity of gymnosperm seeds to environmental conditions, the reproductive efficiency of gymnosperms is expected to decline under warming and drying trends, exposing them to a high risk of extinction (Umair et al. 2023; Qi et al. 2023). *Pinus dabeshanensis* is sensitive to climate warming. Previous studies have shown that, since the Holocene, rising global temperatures have led to a substantial reduction in the species' suitable range and a sharp decline in population size (Bova et al. 2021; Yan et al. 2024). It is therefore crucial to intensify the assessment of the population dynamics of *P*. *dabeshanensis* under future climate change and to formulate appropriate conservation strategies.

### 4.2 Improving In Situ Conservation by Enhancing the Nature Reserve Network and Habitat Management

In situ conservation of threatened gymnosperms should be targeted and requires improvement. Previous studies have shown that, since the Cenozoic, gymnosperms have declined in many regions due to competition with angiosperms (Biffin et al. 2012; Cun and Wang 2015). Our observations indicate that once human disturbance is eliminated, angiosperms tend to outpace the growth and reproduction of *P. dabeshanensis*, leaving its regeneration under serious threat. The in situ conservation practices implemented by the Hubei Dabie Mountain National Nature Reserve demonstrated that appropriate habitat management can be beneficial for seed germination and seedling growth in natural populations of *P*. *dabeshanensis*, including enhancing habitat heterogeneity through the regulation of understory vegetation and improving light, temperature, and soil nutrient conditions within the forest through thinning and selective felling (Wu et al. 2018; Zhang et al. 2018; Feng et al. 2023; Zhou 2024; Jiao et al. 2025). However, high‐intensity interventions in the understory can cause vegetation damage and habitat fragmentation, posing a threat to wildlife (Li et al. 2006, 2013; Li and Li 2011). The spatial scale, frequency, and intensity of management measures still require optimization, but standardized protocols for existing conservation measures have yet to be established. However, implementing moderate habitat management whilst safeguarding ecological balance and establishing standardized conservation practices should enable the endangered species to survive.

More than half a century ago, China established nature reserves to protect the natural populations of endangered species from anthropogenic disturbances and promote the recovery of endangered species in their natural habitats. They indeed played a positive role at an early stage of population recovery (Zhao et al. 2024). However, the establishment of most nature reserves has not been conducive to the survival of gymnosperms. Gymnosperms rely on specific mechanisms for survival and regeneration (Richardson 2000; Burrows 2021), with fire being a key driver shaping their evolution (He et al. 2016). Currently, nature reserves are vigorously promoting fire prevention, which is accelerating community succession and interspecific competition, thereby posing a threat to gymnosperm groups that are at a competitive disadvantage (Bond 1989). This phenomenon has been confirmed in *Cycas panzhihuaensis* , *Cathaya argyrophylla*, *Abies beshanzuensis*, and *P*. *dabeshanensis* (Fu 1992; Xie and Chen 1999; Wang et al. 2012; Xiang 2015; Xiao et al. 2020; Gao et al. 2025). This suggests that it is necessary to conduct targeted habitat management and assisted reproduction based on the species' biological and ecological characteristics in order to effectively conserve endangered gymnosperms even if they are protected within nature reserves. Therefore, we urge management authorities to introduce legal amendments that encourage reserve managers to implement targeted conservation measures to improve the conservation effectiveness of gymnosperm species.

Protocols for pest and disease control of *P*. *dabeshanensis* have not been formulated. The type tree in the Anhui Yaoluoping National Nature Reserve is dying because of pests and diseases. Officials of the nature reserve have adopted different measures to protect the tree from death, but it appears that their actions have not been effective. Currently, pine wilt disease (PWD) is causing widespread pine mortality in southern China, with most pine populations facing severe threats (Mota and Vieira 2008; Huang 2021; Zhang et al. 2022). Since its first appearance in 1982 in Nanjing of Jiangsu, PWD has spread rapidly across tropical and subtropical regions of China, causing widespread death of *Pinus massoniana* and resulting in significant ecological and economic losses (Yin et al. 1988; Mota and Vieira 2008; Guo 2022; Li et al. 2025). *P. dabeshanensis* is facing a severe threat from PWD because it is distributed sympatrically with its congeneric relative *P. massoniana* . Therefore, establishing a pest and disease monitoring network and formulating pest and disease control plans are of paramount importance for the conservation of pines in the Dabie Mountains and elsewhere.

The genetic diversity of *P*. *dabeshanensis* is not completely in situ protected. Currently, over 50% of the genetic diversity in natural populations is found outside the established nature reserves. Notably, the Dawanggou population—which serves as its core wild population—has not yet been incorporated into the national nature reserve protection system. The area is overgrown with shrubs, which encroach on the space occupied by the seedlings and cause severe natural dieback of branches in the middle and lower parts of mature trees and a reduced ability to withstand storms and snow damage (Xu 2016; Xiang et al. 2017). Furthermore, the area is also frequently subject to human activities such as unauthorized digging and destruction; these threats may all exacerbate the loss of genetic diversity within this population. Therefore, to fully preserve the genetic diversity of this species, active conservation efforts should be undertaken for the Dawangou population. Where necessary, it should be incorporated into a nature reserve for standardized management, thereby providing high‐quality population resources for subsequent targeted conservation measures such as ex‐situ conservation, artificial propagation and population reintroduction.

### 4.3 Conducting Artificial Propagation and Reintroduction to Improve Conservation Effectiveness

Sexual reproduction directly determines the capacity for population regeneration and evolutionary potential (Xu and Zang 2022). Our findings show that, although the artificial propagation of *P. dabeshanensis* was initially successful, some shortcomings still remain. Firstly, the seeds of *P*. *dabeshanensis* used for propagation were single‐sourced, all provided by the local farmer of Miaodaoshan Village (Qingzhong Ye), and the mother trees were selected at random without consideration of genetic diversity. This has limited the maintenance and enhancement of genetic diversity, thereby hindering the long‐term adaptation of reintroduced populations. Secondly, natural pollination is inefficient and seed setting rates are rather low in *P*. *dabeshanensis*; for example, none of the seeds collected in 2023 germinated. Thirdly, current propagation methods are relatively limited, with nurseries primarily relying on seedling propagation from seed, while ignoring asexual propagation such as grafting and cuttings. Fourthly, due to a lack of dedicated funding, the conditions in different nurseries may be far from ideal, leaving seedlings vulnerable to environmental stresses such as low temperatures. For instance, during the winter of 2023, over 20% of the seedlings of *P*. *dabeshanensis* in Miaodaoshan Village died, resulting in a major loss of seedlings and saplings. Assisted propagation would work more efficiently when these issues are resolved. Furthermore, existing research has confirmed that rootstock grafting can be successfully applied in the artificial propagation of this species (Sun et al. 1989); consequently, grafting should be adopted as a supplementary measure. Finally, dedicated funding for the Dabie Mountain five‐needle pine must be increased to enable more effective conservation efforts for this species.

Species reintroduction is recognized as a promising conservation tool to halt and reverse plant extinction or decline (Fenu et al. 2023). Although the nature reserves have achieved some successes and accumulated experience in the reintroduction of *P. dabeshanensis*, several shortcomings should be pointed out. Firstly, habitat selection should be based on research and fully consider the biological and ecological characteristics of *P. dabeshanensis* including elevation, light availability, soil properties, and species competition. Secondly, the attempts at reintroduction of *P. dabeshanensis* should be planned and coordinated. Thirdly, most reintroduced populations suffer from inadequate post‐reintroduction management and monitoring, leading to pronounced fluctuations in survival rates. The survival rate of the reintroduced population in Hubei exceeded 80%. In contrast, of the plants reintroduced to the Liankang Mountain National Nature Reserve, only 10% survived due to a lack of subsequent management which led to intense competition for resources, suppressed growth, and high mortality. The accumulated experience shows that the reintroduced saplings require assisted management, including removal of competitive species, protection against frost and drought, and pest/disease control. Furthermore, long‐term monitoring is essential to track the growth and adaptation of the reintroduced plants, ensuring their growth, reproduction, and natural regeneration. People practising reintroduction should work together with researchers to formulate the best methods of reintroduction and develop a standardized technical protocol for the future reintroduction practices of *P*. *dabeshanensis*.

### 4.4 Optimizing Ex‐Situ Conservation to Improve Conservation Effectiveness

As a vital measure to combat plant extinction, ex‐situ conservation has been widely employed globally to protect rare and endangered plant species (Mounce et al. 2017). Despite some efforts, ex‐situ conservation of *P*. *dabeshanensis* is still in its infancy. Initially, life cycle completion was considered as the primary indicator for the success of ex‐situ conservation (Sharrock and Jackson 2016), referring to the completion of a species from seeds to seeds under cultivated conditions. Currently, ex‐situ collections of *P*. *dabeshanensis* consist mainly of seedlings and saplings, with a relatively long term required before seed production. Secondly, the existing ex‐situ efforts inadequately cover the genetic diversity of the species' natural distribution range. Ex‐situ populations exhibit lower genetic variation than their wild counterparts, with certain private alleles of natural populations missing in ex‐situ populations (Xiang 2015). This indicates that the ex‐situ collections do not sufficiently represent the species' full genetic diversity. It is therefore necessary to systematically assess the genetic diversity of natural populations to guide the establishment of ex‐situ conservation populations. Thirdly, *P*. *dabeshanensis* also suffers from incomplete geographic representation in seed banks. The Germplasm Bank of Wild Species in Southwest China collected seeds only from the Dawanggou population of Yuexi County in Anhui, leaving populations from the Hubei Dabie Mountain National Nature Reserve, the Henan Dabie Mountain National Nature Reserve, and the Anhui Jinzhai Tianma National Nature Reserve unsampled. Fourth, current ex‐situ conservation is limited to seed banks, lacking complementary approaches such as pollen and tissue culture preservation. Finally, coordination among botanical gardens, seed banks, and germplasm repositories remains inadequate; to achieve this goal, a comprehensive data management system for tracking ex‐situ materials needs to be established.

### 4.5 Strengthening Genetic Diversity Research and Its Application to Support Conservation Measures

Genetic diversity is important for species to maintain their evolutionary potential and adapt to environmental changes (Sjöqvist 2022; Xu et al. 2015). Previous studies have shown that *P. dabeshanensis* exhibits a moderate level of population genetic diversity, with 40.94% of its genetic variation found between populations (Xiang 2015; Xiang, Zhang, et al. 2015). This may be related to geographic isolation, as the fragmented distribution pattern, narrow habitats, and limited seed dispersal hinder genetic exchange between populations. For instance, the Dawanggou population has few understory seedlings due to inbreeding depression coupled with intense interspecific competition, leading to a declining trend of population size (Xiang 2015; Xiang, Wu, et al. 2015). By reducing the effective population size and increasing homozygosity of deleterious alleles, these factors undermine the evolutionary potential of the populations (Liu et al. 2014).

Genetic diversity also provides a vital basis for the scientific formulation of strategies for in situ conservation, ex‐situ collection, artificial propagation and reintroduction practices (Song et al. 2025). The Kunming‐Montreal Global Biodiversity Framework explicitly called for enhanced protection of genetic diversity in biodiversity conservation (CBD 2022). Due to a lack of systematic genetic data, the current germplasm sampling and collection plan for *P. dabeshanensis* is still impracticable. The germplasm of *P*. *dabeshanensis* for ex‐situ conservation comes from a few individuals of the Dawanggou population and does not encompass the full genetic representation in its natural populations. The random sampling approach employed during germplasm collection has resulted in ex‐situ populations generally exhibiting significantly lower genetic diversity than their wild counterparts; this is a common global issue in the conservation of endangered plants (Xiang 2015; Wei and Jiang 2020). To improve the genetic representation, it is necessary to conduct a systematic survey of the genetic diversity within its natural populations, so as to guide future conservation actions of *P*. *dabeshanensis*. Based on the genetic diversity, the population sampling scope should be refined to collect germplasms representing the species' full genetic diversity spectrum. These well‐represented germplasms should then be propagated in botanical gardens and nurseries to establish a representative ex‐situ population. Furthermore, genetic diversity serves as an important criterion for planning both in situ conservation and reintroduction.

## 5 Conclusion

In this study, we conducted the most comprehensive conservation effectiveness assessment of the endangered *P*. *dabeshanensis* to date, based on field investigations and a literature survey. We determined that this pine species is threatened due to its extremely small population size, aging demographic structure, low natural reproductive efficiency, limited seed dispersal capacity, and intense interspecific competition. A preliminary network of in situ conservation has been established to protect the natural populations from human disturbances, but it is incomplete and unevenly implemented, the largest natural population falling outside any known nature reserve and lacking effective management. Habitat management has been demonstrated to be effective in population recruitment in Hubei, but this successful experience has not been formulated into a protocol to guide conservation practices in other populations. This pine species has been planted in seven botanical gardens and the Germplasm Bank of Wild Species in Southwest China, but the ex‐situ populations are single‐sourced and do not represent the full genetic diversity of the species. Propagation nurseries were established in Henan, Hubei and Anhui, but the facilities are simple, and assisted pollination should be conducted to improve the pollination rates and increase seed production. Reintroduction has been practised in different places and valuable knowledge has been accrued, but these have not been formulated as guidelines. We suggest developing a strategic plan for an integrated conservation of this pine species and formulate technical protocols and guiding manuals to improve the effectiveness of conservation. We also encourage coordinated conservation efforts among different institutions and nature reserves, including research, in situ conservation, ex‐situ conservation, propagation, and reintroduction. This study provides a systematic evaluation of the conservation effectiveness of *P*. *dabeshanensis*, identifies conservation gaps, and proposes an integrated conservation strategy and coordinated efforts. The conservation problems of this pine species are common to many other threatened gymnosperm species. As a result, our study also offers a model for integrated conservation planning of other endangered gymnosperms.

## Author Contributions

**Yan Xu:** data curation (equal), investigation (equal), writing – original draft (equal). **Chao Tan:** investigation (equal). **David Kay Ferguson:** writing – review and editing (equal). **Yong Yang:** conceptualization (equal), investigation (equal), writing – review and editing (equal).

## Funding

This work was supported by the National Key Research Development Program of China [2023YFF0805800].

## Ethics Statement

This study was conducted in accordance with local legislation and the Convention on the Trade in Endangered Species of Wild Fauna and Flora.

## Consent

The authors have nothing to report.

## Conflicts of Interest

The authors declare no conflicts of interest.

## Data Availability

All data used in the study are included in this paper and are available.
